# Translating Expertise: The Librarian’s Role in Translational Research

**DOI:** 10.5195/jmla.2018.349

**Published:** 2018-01-02

**Authors:** Elizabeth Connor

More than ten years ago, the National Institutes of Health (NIH) introduced its Clinical and Translational Science Award (CTSA) designation for institutions focused on fast-tracking research breakthroughs from laboratory bench to bedside. This volume includes case studies from librarians and other professional staff who are affiliated with CTSA consortium institutions and provides a wealth of first-hand information about supporting team science through collection development, instruction, data management, and collaborative partnerships. Current and prior author affiliations of this book’s contributors include public and private institutions such as Duke University, Howard University, Indiana University School of Medicine, New York University School of Medicine, Northwestern University, University of Florida, University of Massachusetts, University of Michigan, University of Utah, University of Washington, and Washington University School of Medicine.

The work is organized into seventeen chapters divided into five broad sections: “Basic and Clinical Science,” “Education and Community Engagement,” “Networks and Connections,” “Infrastructure,” and “Evaluation.” Highlights include the background of translational science (chapter 1), engaging community partners (chapter 6), developing a networking app (chapter 10), complying with public access policy (chapter 12), and measuring publication impact (chapter 17). In between, other excellent chapters provide nuts-and-bolts descriptions of what worked and did not work at specific CTSA sites.

Each chapter is enhanced with text boxes, illustrations, and pertinent bibliographic citations. Some of the black-and-white images are blurry. The index is a scant two pages. A glossary of terms would have been useful for readers who are unfamiliar with this subject matter.

That said, this book is recommended for librarians who support CTSA efforts. Experienced and entry-level medical librarians alike will find much here to adapt to their individual work settings and to reinforce the value of their current and future efforts.

